# Small Vessel Disease on Neuroimaging in a 75-Year-Old Cohort (PIVUS): Comparison With Cognitive and Executive Tests

**DOI:** 10.3389/fnagi.2018.00217

**Published:** 2018-07-16

**Authors:** Ruta Nylander, Lena Kilander, Håkan Ahlström, Lars Lind, Elna-Marie Larsson

**Affiliations:** ^1^Department of Surgical Sciences, Radiology, Uppsala University, Uppsala, Sweden; ^2^Department of Public Health and Caring Sciences, Geriatrics, Uppsala University, Uppsala, Sweden; ^3^Department of Medical Sciences, Uppsala University, Uppsala, Sweden

**Keywords:** small vessel disease, magnetic resonance imaging, perfusion, white matter hyperintensities, lacunar infarct, cognitive tests

## Abstract

**Background and Purpose**: Signs of small vessel disease (SVD) are commonly seen on magnetic resonance imaging (MRI) of the brain in cognitively healthy elderly individuals, and the clinical relevance of these are often unclear. We have previously described three different MRI manifestations of SVD as well as cerebral perfusion in a longitudinal study of non-demented 75-year-old subjects. The purpose of the present study was to evaluate the relationship of these findings to cognition and executive function at age 75 and changes after 5 years.

**Methods**: In all, 406 subjects from the Prospective Investigation of the Vasculature in Uppsala Seniors (PIVUS) study were examined with MRI of the brain at age 75 years. Two-hundred and fifty of the subjects were re-examined 5 years later. White matter hyperintensities (WMHs) and lacunar infarcts (LIs) were assessed on both occasions, but microbleeds (MBs) and perfusion only at age 75. Cognitive function was screened by the Mini Mental State Examination (MMSE). Trail Making Test A and B (TMT-A and TMT-B) were performed at baseline and at follow-up at age 80.

**Results**: At baseline, 93% performed >27 points in the MMSE. The TMT-B at age 75 was significantly related to WMH visual scoring after adjustment for sex, education and cerebrovascular disease risk factors (+80 s (95% CI 0.3–161 s), *P* < 0.05 for grade 2–3 vs. grade 0). Neither MMSE nor TMT-A was significantly related to WMH scoring. There was no relation between any test performance and WMH volume, white matter volume, number of MBs or brain perfusion at age 75. Subjects who had sustained a new LI (*n* = 26) showed a greater increase of the time to perform TMT-A at the 5-year follow-up (+25 s vs. +4 s in LI-free subjects, *P* = 0.003). Changes in MMSE or TMT-A and -B test performance between ages 75 and 80 were not related to changes in WMH scoring or volume during the 5 years follow-up, or to brain perfusion at age 75.

**Conclusion**: In this cognitively healthy community-based population, moderate-severe WMHs and incident LIs on brain MRI in individuals aged 75–80 years were associated with a mild impairment of processing speed and executive function.

## Introduction

Cerebral small vessel disease (SVD) is a common process in the ageing brain, which affects small vessels, including arterioles, capillaries and small veins. SVD predicts stroke, cognitive impairment and depression, and is believed to contribute to approximately 45% of all dementia disorders (Wardlaw et al., 2013b). Multiple and complex pathogenetic mechanisms cause ischemic changes, enlarged perivascular spaces, cerebral microbleeds (MBs) and brain atrophy (Wardlaw et al., 2013a). The resulting brain parenchymal changes detectible by neuroimaging can be regarded as markers of SVD (Pantoni, 2010; de Guio et al., 2016). On magnetic resonance imaging (MRI), these markers are seen as white matter hyperintensities (WMHs) of presumed vascular origin, small subcortical infarcts, lacunar infarcts (LIs), cerebral MBs, prominent perivascular spaces (PVS) and cerebral atrophy (Pantoni, 2010; Wardlaw et al., 2013a).

WMHs are seen on MRI as focal or confluent/more extensive signal changes. They may be located in the periventricular and/or subcortical white matter. LIs evolve into small (3–15 mm) cavities/lacunes in the deep gray or white matter (Wardlaw et al., 2013a). MBs, due to small intramural and perivascular hemeorrhages, are seen on susceptibility-weighted MRI due to residual hemosiderin in macrophages located around small vessels (Pantoni, 2010; Wardlaw et al., 2013b). Perivascular spaces are fluid-filled spaces around small vessels and are detected on MRI when they are enlarged. Brain atrophy that occurs with ageing can be general or focal and has been shown to be associated with the severity of SVD (Wardlaw et al., 2013a).

MRI provides objective information not only on manifestations and progression of changes in the brain parenchyma, but it also allows evaluation of cerebral perfusion. Atherosclerosis and non-atherosclerotic diffuse atheropathy in SVD may produce chronic hypoperfusion, which progresses with increasing age (Pantoni, 2010; Chutinet et al., 2012). Several studies show an association between decreased cerebral perfusion and cognitive decline (Leeuwis et al., 2016; Lou et al., 2016; Zlatar et al., 2016).

Cognitive impairment due to vascular or neurodegenerative dementia disorders develops slowly over several years or decades. In early stages, a subtle cognitive impairment may be reported by the patient or family members but is not always detectable by cognitive tests. The Mini Mental-State Examination (MMSE) is the most widely used cognitive screening instrument, measuring mainly verbal and memory functions. The Trail Making Tests A and B (TMT-A, TMT-B) examine executive functions, including the ability to maintain attention, speed of processing and set-shifting/mental flexibility, mainly reflecting subcortico-frontal functions and cerebrovascular lesions (Conti et al., 2015).

Signs of SVD are often seen on magnetic imaging (MRI) of the brain in cognitively healthy elderly and are highly age-related (Ylikoski et al., 1995; Wardlaw et al., 2015). On a group basis, these changes are associated with lower cognitive and executive performance speed, as shown in several previously reviewed studies of cohorts of different sizes (Debette et al., 2010; Kloppenborg et al., 2014; Wardlaw et al., 2015). One of these studies reviewed 33 cross-sectional and 14 longitudinal studies focused on WMH and cognition (Kloppenborg et al., 2014). However, the clinical role of these imaging findings is often difficult to translate to the individual patient. An association between progression of SVD and decline of general cognitive function and processing speed has also been reported (Debette et al., 2010; Kloppenborg et al., 2014). However, longitudinal results from previous studies are not clear-cut due to different selection of the subjects: many patients in cohorts from Memory Clinics will decline over time; other cohorts have subjective cognitive impairment; some cohorts are healthy community-based samples (Debette et al., 2010; Kloppenborg et al., 2014) others have a wide age range. Studies on the relation between SVD and cognition, including not only WMH but also infarcts and MBs in stroke- and dementia-free subjects between age 75 and 80 years are sparse.

We have previously described three different MRI manifestations of SVD as well as cerebral perfusion in a longitudinal study of non-demented 75-year-old subjects (Nylander, 2017; Nylander et al., 2018). The aim of the present study was to evaluate how cognitive performance on MMSE and TMT-A and -B is related to the WMH, LI, MBs and cerebral perfusion on MRI in a prospective community-based population of cognitively healthy individuals of the same age. We also assessed the evolution over 5 years.

## Materials and Methods

### Ethics Statement

The Regional Ethical Review Board in Uppsala, Sweden, approved the study and all subjects provided written informed consent.

### Study Population

Within the Prospective Investigation of the Vasculature in Uppsala Seniors (PIVUS) study, 406 subjects at the age of 75 years were randomly selected to be examined with MRI of the brain. The subjects had been recruited from the population register of the municipality. MRI was repeated after 5 years in 250 of the 406 subjects who agreed to participate in a follow-up study. Uppsala University Hospital and the primary care in Uppsala County use the same electronic medical records system. All available charts from out-patient and in-patient care of the included individuals were reviewed until each subject reached the age of 80 and all dementia diagnoses were noted. Clinical diagnoses of vascular dementia (VAD), Alzheimer’s disease (AD), Parkinson’s disease dementia (PDD) and unspecified dementia (UNS; cases lacking sufficient information) were verified by one of the authors (Ronnemaa et al., 2011).

### Morphological MRI

MRI of the brain had been performed on a 1.5 Tesla MRI system (Gyroscan Intera, Philips Medical Systems, the Netherlands). At ages 75 and 80 years, the protocol included sagittal 3-dimensional (3D) T1-weighted (TR 8.6 ms, TE 4 ms, slice thickness 1.2 mm, pixel size 0.94 × 0.94 mm) and transverse proton density and T2-weighted turbo spin echo images (TR 3000 ms, TE 21 and 100 ms, slice thickness 3 mm, pixel size 0.94 × 0.94 mm) as described previously (Nylander et al., 2015). In the 75-year-old cohort, a T2*-weighted sequence for evaluation of MBs was also performed.

**Figure 1:**
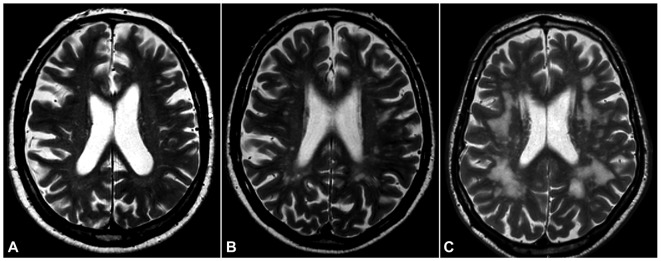
White matter hyperintensities (WMHs). T2-weighted magnetic resonance imaging (MRI) of the brain in three of our subjects, showing three grades of severity according to the Leukoaraiosis And DISability (LADIS) visual scoring scale (modified Fazekas scale): **(A)**, mild; **(B)**, moderate; **(C)**, severe.

WMHs were scored using the visual Leukoaraiosis And DISability (LADIS) rating scale, which is a modification of the widely used Fazekas scale (Inzitari et al., 2009). The scale has three grades where grade three indicates the most severe changes (Figure 1). LIs were defined as hypointense foci (3–15 mm size) on 3D T1-weighted images (Nylander et al., 2018).

### Perfusion MRI

Cerebral perfusion MRI was performed using DSC MRI with contrast agent injection in the 75-year-old subjects and analyzed in our previous study. (Nylander, 2017; Nylander et al., 2018). Perfusion MRI was not repeated in the 80-year-old subjects due to patient age and potential risk of contrast-induced nephropathy.

### Cognitive Tests

The MMSE, the TMT-A and TMT-B were administered at baseline and follow-up.

The MMSE includes 11 questions, requires only 5–10 min to perform, and is a composite test mainly of memory and verbal functions (Folstein et al., 1975). The TMT-A requires subjects to draw lines connecting the numbers 1–25 unevenly distributed on a sheet of paper, as fast as possible and provides information on visual search and speed of processing (Tombaugh, 2004). The TMT-B is more complex than the TMT-A since the subject is asked to draw lines as fast as possible alternating between numbers (1–13) and letters (A–L) in the right order, testing the ability to shift attention and action, i.e., mental flexibility or executive functions. The score on each part represents the time in seconds required to complete the task, and a higher score means a slower performance. The maximum time was set to 180 s for the TMT-A and to 600 s for the TMT-B.

### Cardiovascular Risk Factors/Markers

Recordings of cerebrovascular risk factors were available at both ages (Nylander et al., 2015). Systolic and diastolic blood pressure, smoking, diabetes mellitus, serum total cholesterol and body mass index (BMI) were used for adjustment of statistical analyses.

### Data Analysis and Visual Image Evaluation

WMHs were assessed qualitatively (visual scoring grade 1–3, where 3 is most severe) and quantitatively (semi-automatic volume calculation), and brain tissue segmentation was performed automatically as described previously (Nylander et al., 2018). As previously described LIs and MBs were assessed visually, and the perfusion maps were evaluated with regard to regional and supratentorial rCBF (Nylander et al., 2018).

The WMH volume was analyzed at ages 75 and 80 years using the CASCADE software (ki.se/en/nvs/cascade; Damangir et al., 2012; Cover et al., 2016; Nylander et al., 2018).

CBF maps were calculated, and regional values relative to the cerebellum were used for the analyses (Nylander et al., 2018).

### Statistical Analysis

STATA14 (Stata Inc., College Station, TX, USA) was used for calculations. *P* < 0.05 was regarded as significant.

The relationships between the cognitive function tests and markers of cerebral SVD at age 75 (cross-sectional) were evaluated with Tobit regression (since all three tests were censored at high levels). The models were adjusted for sex, education level, baseline systolic blood pressure, HDL and LDL cholesterol, BMI, smoking and diabetes.

The relationships between changes in the cognitive function tests and changes in markers of cerebral SVD over 5 years (longitudinal) were evaluated with mixed models with a random intercept. Also, these models were adjusted for sex, education level, baseline systolic blood pressure, HDL and LDL cholesterol, BMI, smoking and diabetes.

## Results

An overview of cardiovascular risk factors, imaging findings and cognitive tests at age 75 and 80 are shown in Tables 1, 2.

**Table 1:** Description of the population at baseline.

	Data at age 75	
Characteristics	*N*	Mean (SD)
SBP (mmHg)	406	148.1 (18.8)
DBP (mmHg)	406	76.0 (9.8)
Blood sugar (mmol/L)	406	5.2 (1.4)
Cholesterol (mmol/L)	406	5.4 (1.1)
WMH score	406	1.5 (0.7)
GM volume (ml)	404	564.8 (49.5)
WM volume (ml)	404	465.3 (58.7)
WMHs volume (ml)	396	10.5 (5.2)
Number of microbleeds	406	0.5 (3.0)
MMSE (points)	398	29 (1.4)
TMT-A (s)	394	57 (22)
TMT-B (s)	392	170 (117)

*SBP, systolic blood pressure; DBP, diastolic blood pressure; WMHs, white matter hyperintensities; GM, gray matter; WM, white matter; MMSE, mini mental state examination test; TMT-A, trail making test A; TMT-B, trail making test B*.

**Table 2:** Description of the population who returned for follow-up after 5 years.

	Data at age 75		Data at age 80	
Characteristics	*N*	Mean (SD)	*N*	Mean (SD)
SBP (mmHg)	252	147.8 (18.8)	251	146.9 (18.4)
DBP (mmHg)	252	75.5 (10.0)	251	73.5 (9.4)
Blood sugar (mmol/L)	252	5.2 (1.4)	248	5.3 (1.6)
Cholesterol (mmol/L)	252	5.5 (1.1)	251	5.2 (1.1)
WMH score	252	1.4 (0.7)	252	1.5 (0.7)
GM volume (ml)	252	568.9 (51.1)	231	554.5 (52.4)
WM volume (ml)	252	466.1 (59.7)	231	453.2 (59.1)
WMHs volume (ml)	246	10.1 (4.7)	229	11.9 (5.7)
Number of microbleeds	252	0.5 (3.0)	n/a	n/a
MMSE (points)	250	28.8 (1.2)	242	28 (1.9)
TMT-A (s)	248	53.8 (17.6)	239	60 (30)
TMT-B (s)	247	142.8 (87.2)	237	150 (77)

*SBP, systolic blood pressure; DBP, diastolic blood pressure; WMHs, white matter hyperintensities; GM, gray matter; WM, white matter; MMSE, mini mental state examination test; TMT-A, trail making test A; TMT-B, trail making test B*.

### Findings at Age 75

At baseline, none of the individuals had a dementia diagnosis, and 93% performed >27 points in the MMSE, i.e., the vast majority had no cognitive impairment (Table 1).

Moderate or severe (grades 2 or 3) WMHs were found in 162 (40%) of the 406 subjects at age 75 (Nylander et al., 2018).

Out of 406 subjects, 89 (22%) had one or more LIs and only a few had cortical infarcts, 56 (14%) had MBs (Nylander et al., 2015).

**Figure 2:**
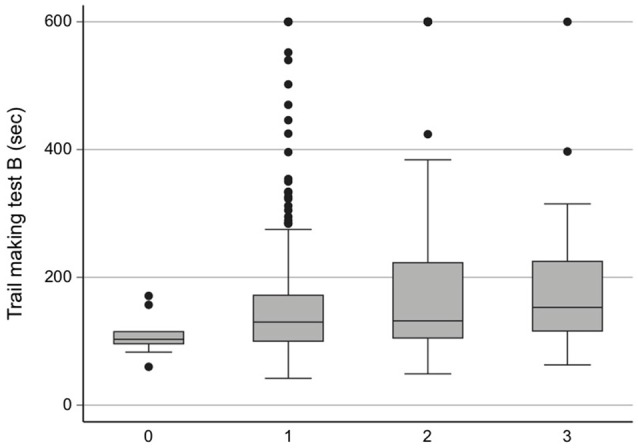
LADIS score vs. Trail Making Test B (TMT-B) results at age 75 years (*n* = 392).

The time to perform TMT-B at age 75 was significantly related to WMH visual scoring after adjustment for sex, education, baseline systolic blood pressure, HDL and LDL cholesterol, BMI, smoking and diabetes (+80 s for WMH grades 2 and 3 vs. grade 0 (95% CI 0.3–161 s), *P* < 0.05; Figure 2). Neither MMSE nor TMT-A was significantly related to WMH scoring. None of the three cognitive tests was significantly related to WMH volume, WM volume, number of MBs, total brain perfusion, WM perfusion or to perfusion in specific brain areas.

### Changes in Cognitive Performance Between Age 75 and Years Compared With Changes of MRI Findings

After 5 years, there was progression of WMH volume, WMH scoring and LIs.

**Figure 3:**
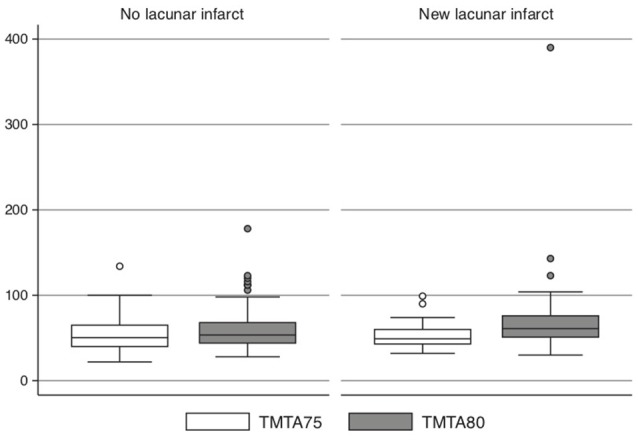
Subjects (*n* = 26) who developed a new lacunar infarct (LI) during the 5-year follow-up compared with those (*n* = 168) without new LI vs. time to perform TMT-A at age 75 and 80.

The 26 subjects who developed a new LI during the 5-year follow-up showed a greater increase of the time to perform TMT-A over 5 years than those who did not develop any LIs (*n* = 224; +25 s vs. +4 s, *P* = 0.003) after adjustment for sex, education level, baseline systolic blood pressure, HDL and LDL cholesterol, BMI, smoking and diabetes (Figure 3 and Table 3). The same trend was seen with regard to the TMT-B (but not the MMSE); however, this trend was not significant (+24 s vs. +2 s, *P* = 0.24, *n* = 172).

**Table 3:** Description of the 26 subjects who gained lacunar infarcts between age 75 and 80.

	Data at age 75		Data at age 80	
Characteristics	*N*	Mean (SD)	*N*	Mean (SD)
SBP (mmHg)	26	156.9 (20.0)	26	154.7 (15.3)
DBP (mmHg)	26	76.4 (11.3)	26	77.1 (8.2)
Blood sugar (mmol/L)	26	4.9 (0.6)	26	5.3 (1.2)
Cholesterol (mmol/L)	26	5.9 (1.1)	26	5.4 (1.1)
WMH score	26	1.7 (0.6)	26	1.7 (0.7)
GM volume (ml)	26	584.1 (40.3)	24	578.2 (44.9)
WM volume (ml)	26	487.4 (44.9)	24	468.6 (44.2)
WMHs volume (ml)	26	11.0 (4.4)	24	14.1 (6.3)
Number of microbleeds	26	0.4 (0.2)	n/a	n/a
MMSE (points)	25	28.4 (1.4)	25	28.2 (2.3)
TMT-A (s)	25	54.3 (16.7)	25	79.6 (69.6)
TMT-B (s)	25	140.9 (103.1)	24	162.1 (82.4)

*SBP, systolic blood pressure; DBP, diastolic blood pressure; WMHs, white matter hyperintensities; GM, gray matter; WM, white matter; MMSE, mini mental state examination test; TMT-A, trail making test A; TMT-B, trail making test B*.

There was no significant association between changes in the MMSE, TMT-A or TMT-B and changes in WMH scoring or volume during 5 years’ follow-up and not to total brain perfusion, WM perfusion or regional perfusion at age 75.

During the 5-year follow-up, clinically diagnosed dementia disorders of different types were found in 18 subjects. Their baseline WMH grades and baseline MMSE are shown in Table 4. Only two of these subjects with incident dementia participated in the follow-up. Non-participants at age 80 performed markedly slower in the TMT-B at baseline (mean 216 s (SD 144) vs. 143 s (SD 87) s in participants).

**Table 4:** Dementia diagnosis, LADIS score and MMSE at baseline in the subjects who were diagnosed with dementia between age 75 and 80 years.

	Data at age 75	
Diagnosis type	LADIS score	MMSE (seconds)
UNS	3	28
VAD	2	28
AD	2	29
AD	2	25
UNS	3	27
AD	2	29
UNS	1	28
VAD	2	27
UNS	2	28
AD	1	24
UNS	1	28
LBD	1	26
AD	3	Not available
UNS	2	27
AD	2	25
UNS	2	26
VAD	1	26
AD	2	26

*UNS, unspecified dementia; VAD, vascular dementia; AD, Alzheimer’s disease; LBD, Lewy body dementia*.

## Discussion

In this community-based population of dementia-free 75 year-old subjects, WMH grade 2–3 was neither associated with impaired performance in global cognition, as measured by the MMSE nor with incident dementia.

WMH grade 2–3 was associated with mildly impaired executive function, as measured by time to perform the TMT-B at baseline. No association was found in the longitudinal data, probably due to a selective loss at follow-up (Table 1).

This finding is consistent with other studies, showing that WMHs mainly affect executive function (Debette et al., 2010; Kloppenborg et al., 2014). Re-evaluation 5 years later did not capture any deterioration in the TMT-A or TMT-B over time associated with WMHs.

During follow-up, incident LIs were associated with slowing of the performance on the TMT-A.

Our results indicate that a majority of healthy elderly subjects with moderate-severe WMHs will not experience any significant decline in cognitive and executive function between age 75 and 80 years. However, our cohort is assumable healthier than a general population. Only 7% had less than 28 points on MMSE at the baseline and during follow–up the incidence of LIs and cognitive decline was low.

### WMHs and Cognition

Several previous studies have described an association between WMHs and cognitive deterioration in high-risk subjects, such as patients referred to a Memory clinic (Benedictus et al., 2015), patients with subjective cognitive impairment (de Groot et al., 2001), or subjects with clinically manifest cerebrovascular disease (Ihle-Hansen et al., 2012), or the LADIS Study where the participants had a higher prevalence of WMHs at baseline (van der Flier et al., 2005; Inzitari et al., 2009; Pantoni et al., 2015).

In patients with subjective cognitive decline, WMHs were reported to be associated with progression of subjective cognitive failures (de Groot et al., 2001), and with a higher risk of incident mild cognitive impairment or dementia (Benedictus et al., 2015). Further, both WMH and cognitive decline are highly age-related. A cognitively intact population of 80 year-olds (Boyle et al., 2016), i.e., older than our subjects, and with high WMH volume, had a 2.7 times higher risk of developing MCI compared to those with low WMH volume. There are several cross-sectional and longitudinal population-based studies on healthy subjects, mainly younger than 80 years, that used a similar study design as ours (Schmidt et al., 2005; Kramer et al., 2007; Smith et al., 2008; van Dijk et al., 2008; Debette and Markus, 2010; Debette et al., 2010; Inaba et al., 2011; Kloppenborg et al., 2014) and the majority, but not all (Schmidt et al., 2005) found an association with impaired global cognition, affecting processing speed and executive function more than memory. However, considering publication bias, all negative studies may not have been published.

The MMSE is the most common screening instrument for cognitive disorders, with focus on verbal function, memory, orientation and calculation (Folstein et al., 1975). The TMT-A measures attention and perceptual speed and the TMT-B examines executive functions, as it also requires cognitive flexibility (Sörös et al., 2015). Impaired performance in these tests is common in patients with a previous TIA or stroke (Ihle-Hansen et al., 2012; Sörös et al., 2015) in contrast to preserved global cognition as measured by the MMSE.

In a recent review, the TMTs were recognized as two of the most frequently used instruments in testing executive function in stroke patients (Conti et al., 2015). Further, we have previously shown in a cohort of stroke-free elderly men that impaired performance in the TMT-B (in contrast to TMT-A or the MMSE) was an independent predictor of subsequent brain infarction (Wiberg et al., 2010). Hence, performance in the TMT-B seems to be a sensitive marker of both clinical overt and subclinical cerebrovascular disease.

The cross-sectional association between WMH grade 2–3 and impaired results in the TMT-B is in agreement with several studies that have shown a relationship between WMH and impaired executive function including processing speed, commonly assessed by the TMTs, the Stroop Test, the Digit span and the Letter Digit Substitution, among others (Prins et al., 2005; Kramer et al., 2007; Wiberg et al., 2010; Benedictus et al., 2015; Conti et al., 2015; Dong et al., 2015; Boyle et al., 2016). Impaired executive function mirrors subcortico-frontal dysfunction and is consistent with the localization of WMHs.

### LI and Cognition

LIs as a part of SVD have previously been reported to be associated with a higher risk of dementia, mainly in individuals with WMHs, cortical atrophy and recurrent strokes (Loeb et al., 1992). In the LADIS study, progression of LIs showed an association with cognitive impairment, mostly in processing speed and executive function (Jokinen et al., 2011). This is in agreement with our study, showing that appearance of new LIs was associated with a slower speed in the TMT-A during the 5-year observation period.

WMHs and LIs were independently associated with a decline of general cognitive function in the LADIS study (Pantoni et al., 2015), which used the MMSE and a modified AD Assessment scale (ADAS) for 633 independently living elderly subjects (van der Flier et al., 2005). Also, the longitudinal evaluation in the LADIS study showed a significantly steeper decline of cognitive function and showed a risk of developing dementia and medial temporal atrophy in patients with a combination of severe WMH and lacunas, independently of age, sex or education (Pantoni et al., 2015).

### MB and Cognition

Cerebral MBs are part of SVD, and the number of MBs increases with age. Recent longitudinal studies showed that MBs have an impact on cognitive impairment, which varies with their location in the brain (van Norden et al., 2011; Poels et al., 2012). In the Rotterdam study, a higher number of MBs was associated with a lower MMSE score and worse performance on tests of information processing speed and motor speed. The presence of lobar MBs was associated with worse performance on tests measuring cognitive function even after adjustments for vascular risk factors and other imaging markers of SVD (Poels et al., 2012). In our study, the number of MBs was not significantly related to cognitive tests at the age of 75, which is not in agreement with these findings. The Rotterdam study stated that the location (lobar) and number of MBs are related to cognitive decline: a higher number of MBs was associated with lower results on a cognitive test (Poels et al., 2012). However, in our sample only 14% (56 of 406 subjects) had MBs, and most of them had less than five MBs. The MRI markers of SVD, WMHs, LIs and MBs have in other studies been shown to be independently associated with cognitive decline and dementia (van der Flier et al., 2005; Jokinen et al., 2011; Østergaard et al., 2016).

### Perfusion and Cognition

Our findings did not show a significant relationship between cerebral perfusion and cognitive functions at age 75, neither in total nor regionally. This is in line with a recent study of non-demented patients showing that cognitive function was stronger associated with white matter integrity than with perfusion (Zhong et al., 2017). In contrast, another study showed an association between reduced cerebral blood flow velocity in the middle cerebral artery, more severe WMH and cognitive impairment (Alosco et al., 2013). In a study of patients with hypertension, higher white matter lesion volume was related to regional decrease of perfusion within areas of WMHs but not to a reduction of general perfusion (van Dalen et al., 2016). Cerebral perfusion can be measured by MRI in different ways, but the dynamic susceptibility contrast (DSC) technique used in our study has so far been most commonly used. However, arterial spin labeling (ASL) perfusion without contrast agent injection and with the possibility to obtain absolute CBF-values is increasingly used, especially for research studies (van Dalen et al., 2016).

### Strengths and Limitations

Our study population was a community-based sample of cognitively healthy 75-year-old subjects and we were able to repeat the MRI scans at the age of 80 in 62%. The study participants in this cohort were of the same age, and this homogeneity means that the confounding effect of age, which is strongly associated with WMHs, was minimized without requiring further adjustment in multivariable regression models.

The relative reduction of the sample size at follow-up was a limitation: 38% was lost due to different diseases and/or unknown conditions, which affects the conclusions.

The observation period was only 5 years. Longer follow-up periods, expanding the age and sample size, may reveal further progression of SVD in association with cognitive decline.

To determine cognitive function, the MMSE was used to get a composite cognitive assessment, and the TMT-A (process speed), and TMT-B (executive function) were used in both cohorts. For further investigations, a wider range of tests on memory, language and executive function with greater sensitivity to early decline would be preferable.

Both quantitative and qualitative techniques for the detection of WMHs have limitations. However, visual rating scales may not always provide the same results as the measurement of absolute lesion volume (van Straaten et al., 2006), and we have used both methods in the present study.

We found no association between rCBF and cognitive tests. The physiological variability of rCBF may be too large to reveal small differences among participants on DSC perfusion. No perfusion study was performed at age 80 years (due to patient age and potential risk of contrast-induced nephropathy), which prevented comparison of perfusion over time. Also, the DSC perfusion method only provides relative values.

Our study focused on MRI manifestations of SVD and their relationship to cognitive impairment. However, synergistic effect between these changes and concomitant diseases including stroke and neurodegenerative disorders may also be of importance.

## Conclusion

Executive function, as measured by the TMT-B, at age 75 was significantly related to WMH score. In the longitudinal part of this study, progression of LIs over 5 years was associated with impaired performance in the TMT-A.

No other significant associations were found but cannot be excluded because of the reduced size of the cohort (250 of the original 406) at follow-up.

Thus, SVD on MRI is not only an incidental finding related to normal ageing. Moderate-severe WMHs or new LIs on MRI of the brain in elderly individuals may be considered potential risk factors for decline in cognitive function. However, according to our results, it shall also be noted that the vast majority of 75 year olds with moderate to severe WMHs will not experience a significant decline in cognitive or executive function during the next few years.

## Author Contributions

LL, HA: substantial contribution to the conception of the work. E-ML and LK: design of the work. RN and EML: acquisition and analysis. LL and LK: interpretation of data for the work. RN: drafting the work. LL, LK, E-ML and RN: revising it critically for important intellectual content. RN, LK, HA, LL and E-ML: final approval of the version to be published and agreement to be accountable for all aspects of the work ensuring the questions related to the accuracy or integrity of any part of the work are appropriately investigated and resolved by all authors.

## Conflict of Interest Statement

The authors declare that the research was conducted in the absence of any commercial or financial relationships that could be construed as a potential conflict of interest.
